# Supplementary figures and images for: Porcn is essential for growth and invagination of the mammalian optic cup
Source: Front Cell Dev Biol. 2022 Oct 31;10:1016182. doi: 10.3389/fcell.2022.1016182 (PMC9661423; doi:10.3389/fcell.2022.1016182)

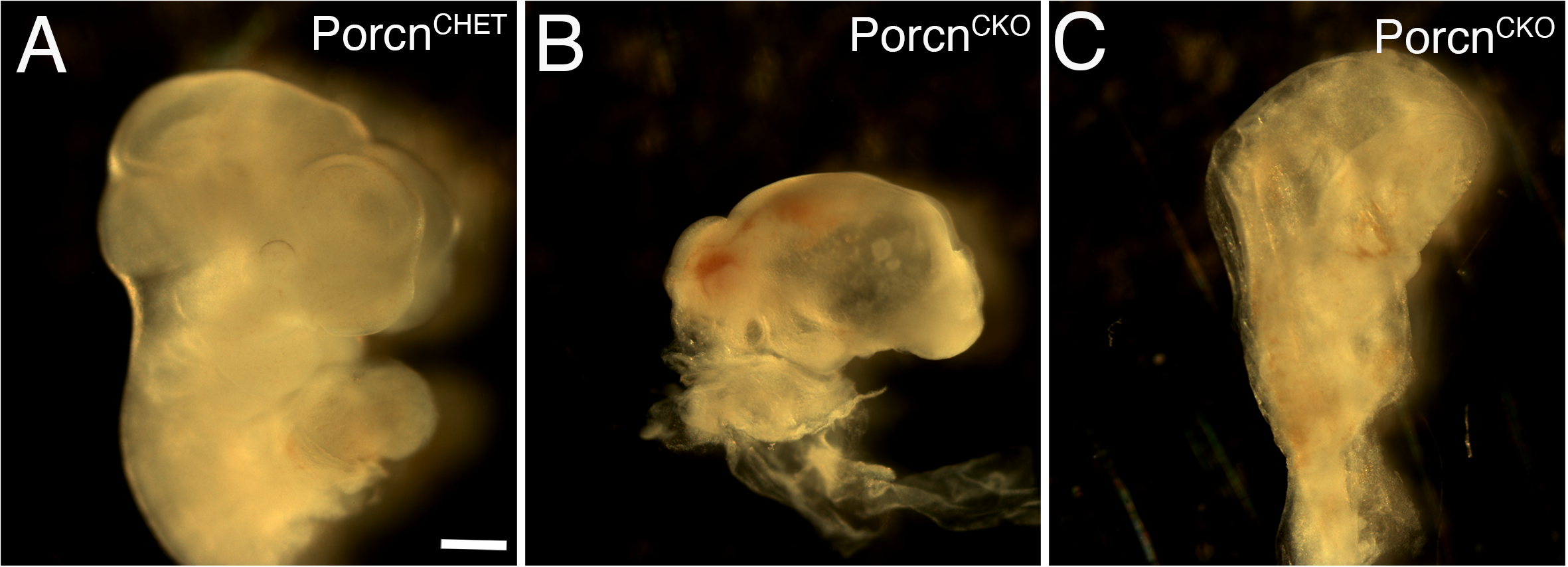

Supplement: Supplementary file 1 [file Image2.TIF]

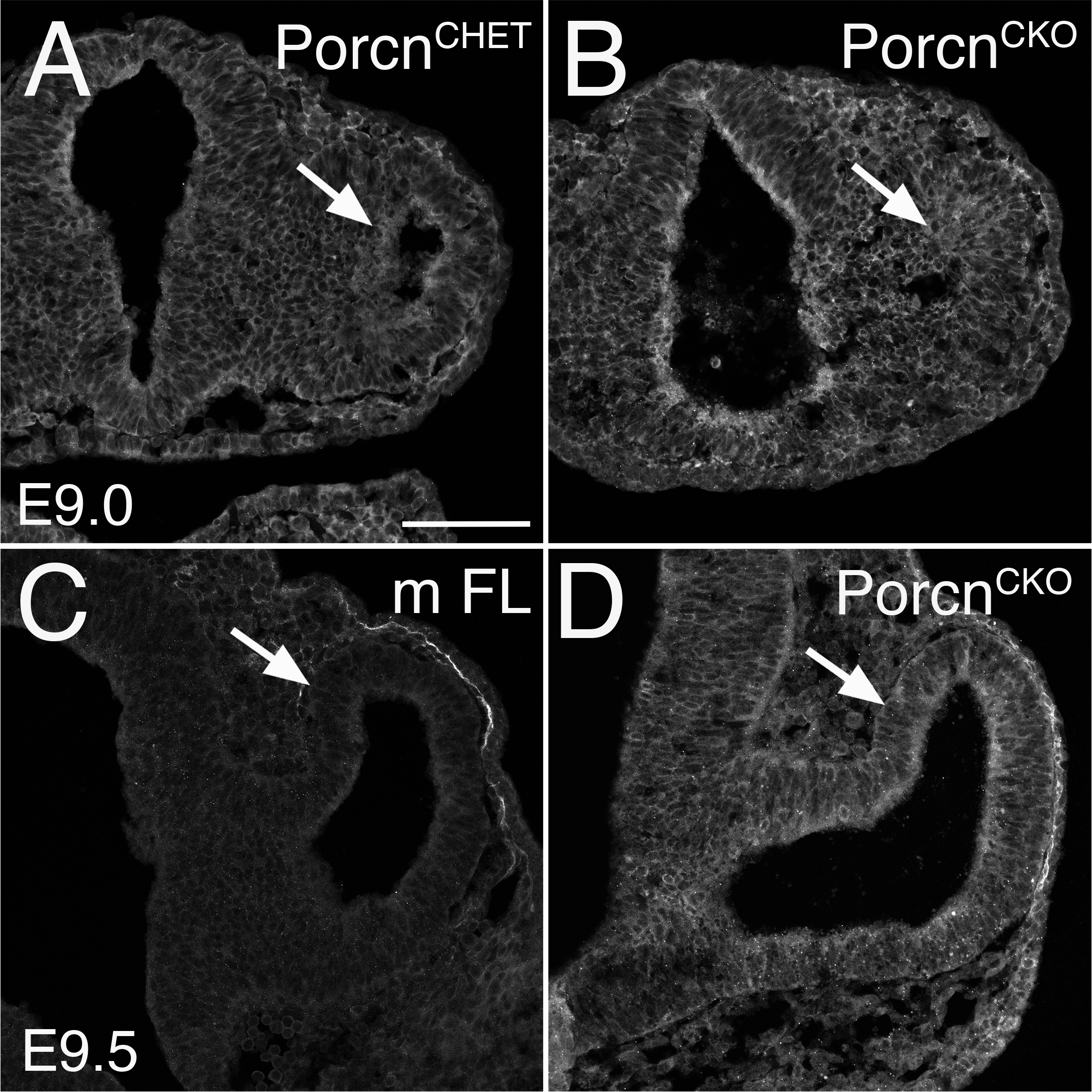

Supplement: Supplementary file 2 [file Image1.TIF]
