# Supplementary material for: Porcn is essential for growth and invagination of the mammalian optic cup
Source: Front Cell Dev Biol. 2022 Oct 31;10:1016182. doi: 10.3389/fcell.2022.1016182 (PMC9661423; doi:10.3389/fcell.2022.1016182)
Supplement: Supplementary file 3 [file DataSheet1.PDF]

Suppl. Table 1: Antibodies used in this study

Primary Antibodies	Dilution	Supplier, Product Number
β-galactosidase	1:5,000	Cappel; MP Biomedicals, Aurora, OH; #55976
LEF1	1:125	Cell Signaling; Danvers, MA; #2230
LHX2	1:300	GeneTex; Irving, CA; #GTX129241
MITF	1:800	Exalpha; Shirley, MA; # X1405M
NR2F2	1:200	Perseus Proteomics; Komaba, Meguro-ku, Tokyo; #PP-H7147-00
OTX2	1:700	R&D Systems; Minneapolis, MN; #AF1979
PAX2	1:800	BioLegend; San Diego, CA; #901001
PAX6	1:500	BioLegend; San Diego, CA; #901301
SIX3	1:300	Rockland Antibodies and Assays;Limerick, PA; #600-401-A26S
VSX2	1:800	Exalpha; Shirley, MA; #X1180P

Secondary Antibodies		
donkey anti-mouse Alexa Fluor®594	1:1,000	Jackson ImmunoResearch; West Grove, PA; #715-585-150
donkey anti-mouse Alexa Fluor647	1:800	Thermo Fisher Scientific; Walham, MA; #A31571
donkey anti-rabbit Alexa Fluor®488	1:1,000	Jackson ImmunoResearch; West Grove, PA; #711-545-152
donkey anti-rabbit Alexa Fluor®594	1:1,000	Jackson ImmunoResearch; West Grove, PA; #711-585-152
donkey anti-goat Alexa Fluor568	1:1,000	Thermo Fisher Scientific; Walham, MA; #A110357
donkey anti-goat TRITC	1:800	Jackson ImmunoResearch; West Grove, PA; #705-025-147
donkey anti-sheep Cy™3	1:800	Jackson ImmunoResearch; West Grove, PA; #713-165-003
